# Culture-Free Enumeration of *Mycobacterium tuberculosis* in Mouse Tissues Using the Molecular Bacterial Load Assay for Preclinical Drug Development

**DOI:** 10.3390/microorganisms10020460

**Published:** 2022-02-17

**Authors:** Dimitrios Evangelopoulos, Carolyn M. Shoen, Isobella Honeyborne, Simon Clark, Ann Williams, Galina V. Mukamolova, Michael H. Cynamon, Timothy D. McHugh

**Affiliations:** 1 Department of Microbial Diseases, UCL Eastman Dental Institute, University College London, London NW3 2PF, UK; 2 UCL Centre for Clinical Microbiology, University College London, London NW3 2PF, UK; i.honeyborne@ucl.ac.uk (I.H.); t.mchugh@ucl.ac.uk (T.D.M.); 3 Veterans Administration Medical Center, Syracuse, NY 13210, USA; shoenc@cnyrc.org (C.M.S.); michael.cynamon@va.gov (M.H.C.); 4 Veterans Health Research Institute, Syracuse, NY 13210, USA; 5 UK Health Security Agency, Porton Down, Salisbury SP4 OJG, UK; simon.clark@phe.gov.uk (S.C.); ann.rawkins@phe.gov.uk (A.W.); 6 Leicester Tuberculosis Research Group, Department of Respiratory Sciences, University of Leicester, Leicester LE1 7RH, UK; gvm4@le.ac.uk

**Keywords:** culture-free quantification, 16S ribosomal RNA, bacterial load, preclinical drug testing, drug combinations, relapse, therapy monitoring, tuberculosis, differentially culturable bacteria

## Abstract

Background: The turnaround times for phenotypic tests used to monitor the bacterial load of *Mycobacterium tuberculosis*, in both clinical and preclinical studies, are delayed by the organism’s slow growth in culture media. The existence of differentially culturable populations of *M.* *tuberculosis* may result in an underestimate of the true number. Moreover, culture methods are susceptible to contamination resulting in loss of critical data points. Objectives: We report the adaptation of our robust, culture-free assay utilising 16S ribosomal RNA, developed for sputum, to enumerate the number of bacteria present in animal tissues as a tool to improve the read-outs in preclinical drug efficacy studies. Methods: Initial assay adaptation was performed using naïve mouse lungs spiked with known quantities of *M. tuberculosis* and an internal RNA control. Tissues were homogenised, total RNA extracted, and enumeration performed using RT-qPCR. We then evaluated the utility of the assay, in comparison to bacterial counts estimated using growth assays on solid and liquid media, to accurately inform bacterial load in tissues from *M. tuberculosis*-infected mice before and during treatment with a panel of drug combinations. Results: When tested on lung tissues derived from infected mice, the MBL assay produced comparable results to the bacterial counts in solid culture (colony forming units: CFU). Notably, under specific drug treatments, the MBL assay was able to detect a significantly higher number of *M. tuberculosis* compared to CFU, likely indicating the presence of bacteria that were unable to produce colonies in solid-based culture. Additionally, growth recovery in liquid media using the most probable number (MPN) assay was able to account for the discrepancy between the MBL assay and CFU number, suggesting that the MBL assay detects differentially culturable sub-populations of *M. tuberculosis*. Conclusions: The MBL assay can enumerate the bacterial load in animal tissues in real time without the need to wait for extended periods for cultures to grow. The readout correlates well with CFUs. Importantly, we have shown that the MBL is able to measure specific populations of bacteria not cultured on solid agar. The adaptation of this assay for preclinical studies has the potential to decrease the readout time of data acquisition from animal experiments and could represent a valuable tool for tuberculosis drug discovery and development.

## 1. Introduction

Tuberculosis (TB) continues to be one of the top ten causes of death worldwide and in 2020 alone more than 1.5 million people died of the disease [1]. Drug-sensitive cases of TB are treated with a combination of four first-line drugs (isoniazid: INH, rifampicin: RIF, ethambutol: EMB, and pyrazinamide: PZA) for 6 months with a cure rate of more than 80% [2]. However, strains of *Mycobacterium tuberculosis* resistant to a single (mono-resistance) or multiple drugs (MDR/XDR-TB) have emerged and spread globally affecting more than half a million patients every year [1]. Drug-resistant TB is an urgent health emergency with higher costs associated with its treatment and cure rates for XDR-TB are below 40%. Since treatment options for drug-resistant TB are limited, there is an urgent need to develop new drugs and drug regimens as well as other therapeutics to tackle the disease, increase cure rates and reduce the treatment course duration. Currently, there are several new drugs as well as new drug regimens under discovery and development for treating TB [3]. One of the recent phase 3 clinical trials reported non-inferiority for a new 4-month regimen containing rifapentine instead of rifampicin and moxifloxacin instead of ethambutol compared to the standard 6-month treatment period providing, for the first time, encouraging data towards treatment shortening [4]. Several other trials are investigating the shortening of the standard treatment as well as new drug combinations for treating both drug-sensitive and drug-resistant TB.

One way to accelerate the drug development pipeline depends on having predictable preclinical models where new drugs and drug combinations are prioritised for clinical trials. The hollow fibre model is the only in vitro model currently endorsed by both Food and Drug Administration (FDA)and the European Medicines Agency (EMA) to take into account pharmacodynamic and pharmacokinetic parameters [5]. Other preclinical studies on drug development rely heavily on animal models and primarily the mouse model of infection where new drug regimens are tested to determine their efficacy in clearing TB infections [6]. In addition to considering the distribution of drugs inside tissues and sites of infection, animal models also involve additional parameters including the immune response and effects of host metabolism.

One of the main endpoints in animal experiments that test the efficacy of a new drug, or a new drug combination is by the enumeration of viable *M. tuberculosis* bacilli at various timepoints post-infection and during treatment initiation. Colony forming units (CFU/mL) from tissue homogenates (lungs, spleen) are counted on agar-based media and compared to untreated controls. The slow doubling time of *M. tuberculosis* requires at least 3 to 5 weeks of incubation at 37 °C for single colonies to appear and be counted. Another concern with the agar-based measurement is potential contamination that might mask the growth of the mycobacteria, making counting impossible, resulting in missing datapoints. In addition, the waxy nature of the mycobacterial cell wall promotes clumping, resulting in the erroneous extrapolation of single colony counts as representative of a single organism. Furthermore, it has been shown before that agar-based methods can reveal different populations of bacteria than liquid-based ones resulting in variable measurements [7,8]. Differentially culturable bacteria, also known as viable but not culturable bacteria and differentially detectable bacteria present could also complicate the actual enumeration of the bacteria obtained from sputum samples and animal tissues [9,10,11,12]. As a result, animal-based data from colony counts could well be an under estimate of the actual bacterial burden present and it could misinform the prioritisation of future drug development strategies [11,13].

Rapid detection of *M. tuberculosis* using molecular assays has been utilised for more than 10 years, with the endorsement of the GeneXpert MTB/RIF assay by WHO in 2010 [14]. For monitoring treatment outcomes, however, the detection of mycobacterial DNA does not correlate with a decline in viable *M. tuberculosis* bacilli during treatment response [15,16]. The evidence suggests that GeneXpert is at best semi-quantitative [17]. For these reasons, we developed and trialled the molecular bacterial load (MBL) assay for enumeration of live TB bacilli from sputum using the detection of 16S ribosomal RNA [18,19]. We have tested this assay in different settings using retrospective samples but also in real time for monitoring treatment responses and managing TB patients [20,21]. Since the test offers culture-free enumeration of bacterial load, we wanted to apply this assay in preclinical settings to accelerate this part of the drug discovery pipeline and to provide more informative outcomes of drug treatment. Our initial comparison of the MBL assay with colony counts and liquid-based growth from mouse tissue infected with *M. tuberculosis* and treated with TB chemotherapy of drug combinations provided encouraging data supporting the use of the MBL assay on tissues in place of culture-based methods [8].

Here we show that the MBL assay can be utilised in the enumeration of bacterial load from infected as well as from drug-treated animal tissues. We have utilised lung tissues from three separate mouse studies evaluating various drugs and drug combinations using different mouse models. In addition, using the MBL assay, information about the sterilising efficacy of a new drug combination can be obtained predicting future regimens that could reduce the treatment duration without the risk of later relapse. In summary, these observations make the MBL assay and the monitoring of 16S rRNA a reliable biomarker for culture-free cell viability in preclinical settings.

## 2. Materials and Methods

All materials were purchased from Sigma unless otherwise stated. Uninfected mouse tissues were obtained from UK Health Protection Agency (UKHSA, Porton Down, Salisbury, UK) BALB/c mice were originally sourced from Envigo, UK and euthanised by cervical dislocation.

### 2.1. Bacterial Strains and Culture Conditions

*M. tuberculosis* ATCC 35801 (strain Erdman) and ATCC 27294 (strain H37Rv) were obtained from the American Type Culture Collection (ATCC) Manassas, VA. *M. tuberculosis* strain H37Rv (ATCC27294) was used for all validation experiments whereas both *M. tuberculosis* strain H37Rv and *M. tuberculosis* strain Erdman (ATCC35801) were used for murine experiments. *M. tuberculosis* H37Rv was grown in Middlebrook 7H9 broth supplemented with 10% oleic acid, albumin, dextrose and catalase (OADC) as standing cultures in a 37 °C incubator. *M. tuberculosis* Erdman was grown in modified 7H10 broth with 10% OADC enrichment and 0.05% Tween 80 on a rotary shaker at 37 °C for 7 to 10 days.

### 2.2. Preparation of the Internal Control (IC)

The internal control is a 1.957 kbp mRNA fragment that was generated as described by Honeyborne et al., 2011 [18]. The concentration of the IC that was used in the assays was 50 ng of RNA in 0.05 mL.

### 2.3. Mouse Experiments

Murine *M. tuberculosis* infection model. Six-week-old female C57BL/6 mice and BALB/c were purchased from Jackson Laboratories, Bar Harbor, ME, and Charles River Laboratories, Wilmington, MA. Mice were maintained within the ABSL-3 at the Syracuse VA Medical Center’s Veterinary Medical Unit, Syracuse, NY. All animal procedures were approved by the Subcommittee for Animal Studies (SAS). Mice were housed in micro-isolator cages (lab products inc. Maywood, NJ, USA) and maintained with water and Prolab RMH 3000 rodent chow (Purina, St. Louis, MO, USA).

*M. tuberculosis* was grown in modified 7H10 broth (pH 6.6; 7H10 agar formulation with agar and malachite green omitted) with 10% OADC (oleic acid, albumin, dextrose, catalase) enrichment (BBL Microbiology Systems, Cockeysville, MD, USA) and 0.05% Tween 80 for 5–10 days on a rotary shaker at 37 °C. The culture was diluted to 100 Klett units (equivalent to 5 × 10^7^ colony forming units (CFU)) per mL (Photoelectric Colorimeter; Manostat Corp., New York, NY, USA). The culture was frozen at −70 °C until using the same modified 7H10 medium. On the day of infection, the culture was thawed, sonicated and diluted to the appropriate concentration in a modified 7H10 medium. The final inoculum size in each experiment was determined by titration, in triplicate, on 7H10 agar plates (BD Diagnostic Systems, Sparks, MD, USA) supplemented with 10% OADC enrichment. The plates were incubated at 37 °C in ambient air for 4 weeks. All mice were infected intranasally after anesthetising with an intramuscular injection of a telazol (45 mg/kg)/xylazine (7.5 mg/kg) cocktail (Lederle Parenterals, Carolina, Puerto Rico and Bayer Corp., Shawnee Mission, Kansas, respectively) and subsequently placing 20 μL of viable *M. tuberculosis* on the external nares of the mouse.

There were three separate infection/treatment studies. In study 1, BALB/c mice were infected with 4.6 × 10^3^ CFU of *M. tuberculosis* H37Rv. One week post-infection, three mice (early controls: EC) were euthanised by CO_2_ inhalation and the lungs were removed to determine the infection load at the start of therapy. Mice were treated daily by oral gavage with a 0.2 mL volume of rifampicin (RIF) or ethambutol (EMB) (Sigma Chemical Co., St. Louis, MO, USA) for 14 days. For dose administration, RIF was dissolved in 20% dimethyl sulfoxide (DMSO) and was administered at 10 mg/kg. EMB was dissolved in 0.25% carboxymethylcellulose and was dosed at 100 mg/kg. There was a group of untreated mice used as a control to determine the infection load at the end of treatment (late controls: LC).

In study 2, C57BL/6 mice were infected with 3.5 × 10^6^ CFU of *M. tuberculosis* Erdman. One week post-infection, mice were euthanised to determine the infection load at the start of therapy. Mice were treated daily by oral gavage with a 0.2 mL volume of isoniazid (INH; Sigma Chemical Co., St. Louis, MO, USA) or sutezolid (PNU-100480 [U480]; NIH, Bethesda, MD, USA) for 14 days. Rifalazil (RZL; Jacobus Pharmaceuticals) was administered 5 days/week for 8 weeks by oral gavage in a 0.2 mL volume. For dose administration, INH was dissolved in distilled water and was administered at 25 mg/kg. U480 was dissolved in 20% DMSO and was dosed at 100 mg/kg. RZL was dissolved in 0.5% hydroxyethylcellulose (HEC) with 10% Tween 80 and was dosed at 60 or 300 mg/kg. There was a group of untreated mice used as a control to determine the infection load after 21 days of infection (late controls: LC). There were parallel groups of mice treated with 8 weeks of RZL at 60 and 300 mg/kg that were observed off therapy for an additional 8 weeks to determine the level of regrowth in the lungs of mice.

In study 3, BALB/c mice were infected with 2.6 × 10^2^ CFU of *M. tuberculosis* Erdman. Eighteen days post-infection, mice were treated with 25 mg/kg INH orally by gavage for three days. After this treatment phase, mice were treated with 60 mg/kg of RZL alone or 60 mg/kg of RZL plus 300 mg/Kg of moxifloxacin (MXF; Carbosynth, San Diego, CA, USA) and 450 mg/kg of pyrazinamide (PZA; Sigma Chemical Co. St. Louis, MO, USA) by oral gavage 5 days/week for 4 weeks. The RZL and RZL combinations were all dissolved in 0.5% HEC (with 10% Tween 80).

All mice were euthanised by CO_2_ inhalation after the completion of therapy. The right lungs were aseptically removed and ground in a sealed tissue homogeniser (IdeaWorks! Laboratory Devices, Syracuse, NY, USA). The left lungs were placed into 3 mL of 5 M Guanidine thiocyanate (GTC) solution containing 0.7% (*v*/*v*) β-mercaptoethanol prior to freezing. The number of viable organisms was determined by serial dilution and titration on 7H10 agar plates with 10% OADC (oleic acid, albumin, dextrose, catalase) or for studies involving treatment with RZL, homogenates were spread on 7H10 agar plates containing 10% OADC and 0.4% charcoal to bind the free drug. Plates were incubated at 37 °C in ambient air for 4 weeks prior to counting.

### 2.4. Most Probable Number (MPN Assay)

The MPN assay was performed in a subset of mouse samples using serial dilutions of the homogenate from the right lungs and growing them in Middlebrook 7H9 broth media [9]. Briefly, MPN assays were performed in quadruple replicates in 48-well microtiter plates (Greiner Bio-One) by diluting 50 μL of mouse homogenate into 450 μL of the medium; MPN counts were calculated with 95% confidence limits following the procedures of the U.S. Food and Drug Administration [22].

### 2.5. MBL Assay

Validation experiments for the use of the MBL assay on tissues were performed using uninfected mouse tissues that were spiked with either internal control RNA and/or *M. tuberculosis* cells at various CFUs/mL.

Uninfected lung tissues or the left lungs of infected animals were submerged into 4 mL of 5 M Guanidine thiocyanate (GTC) solution containing 0.7% (*v*/*v*) β-mercaptoethanol, kept at room temperature for 2 h before being frozen at −80 °C until the total RNA was extracted.

Prior to RNA extraction, mouse tissues were completely thawed at room temperature and were transferred into genteMACS M tubes. Mouse tissues were then homogenised by attaching the genteMACS M tube upside down into the gentleMACS Dissociator running the programme RNA_01. Following homogenisation, 50 ng of the internal control was added to each sample prior to centrifugation at 3000× *g* for 30 min at room temperature (RT) to pellet the bacteria. Total RNA was then extracted from the remaining pellet using the FastRNA™ Pro Blue kit (MP Biomedicals) according to the manufacturer’s instructions. The extracted RNA was then treated for the presence of genomic DNA using TURBO DNA-free™ kits (Life Technologies), quantified using Nanodrop and stored in −80 °C until RT-qPCR was performed.

The expression levels of 16S rRNA and the IC were determined using a duplex reverse transcriptase RT-qPCR. All reagents were from the Quantitect Multiplex RT-PCR NR Kit (Qiagen), except primers and dual-labelled probes, which were synthesised by Eurofins MWG Operon. RT-qPCR parameters were as previously described [18]. MBL assays from *M. tuberculosis*-infected tissues were performed blinded without prior knowledge of the CFU counts.

### 2.6. Statistical Analysis

GraphPad Prism 9 was used for all statistical analysis and data visualisation.

## 3. Results

### 3.1. Adaptation of the MBL Assay to Tissue Homogenates

**Figure 1:**
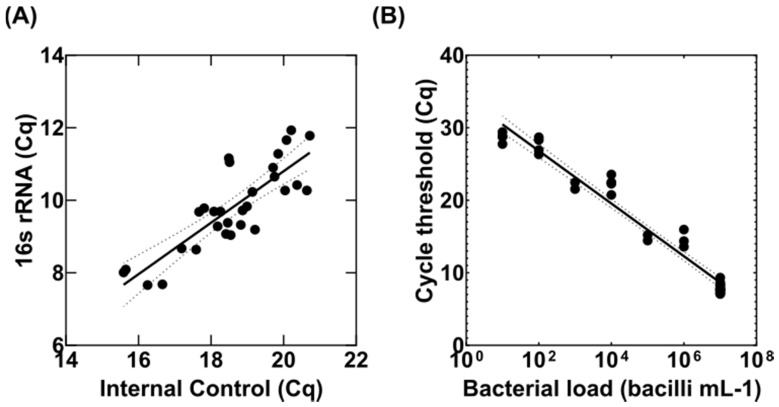
Adaptation and validation of MBL assay in uninfected mouse tissues. (**A**) Correlation between the detection of the internal control (IC) gene and *M. tuberculosis* 16S rRNA in 30 BALB/c mouse tissue samples, spiked with 10^7^ CFUs if *M. tuberculosis* H37Rv and 50 ng of IC. (**B**) The range of qPCR cycle threshold (Cq) from mouse tissues that were spiked with a serial dilution of *M. tuberculosis* H37Rv, fitted to a linear regression model (solid line) with 95% confidence intervals shown as dotted lines. The Cq values were normalised based on the IC as per the correlation graph shown in panel (**A**).

In order to transfer the MBL assay conversion scale for the enumeration of bacterial load in sputum to infected tissues, we first used uninfected mouse lungs that were spiked with various concentrations of *M. tuberculosis* H37Rv and IC RNA. To test the reproducibility of the MBL assay in tissues, we spiked 30 mouse lungs with 10^7^ CFUs/mL *M. tuberculosis* H37Rv and 50 ng of IC. RNA was extracted from these samples following tissue homogenisation and the quantity of 16S rRNA and IC were measured using a duplex RT-qPCR. The R^2^ from a linear regression fit between the quantification cycles (Cq) for 16S rRNA and IC was 0.83 (Figure 1A) closely related to the same R^2^ value of the MBL assay from sputum samples [18]. Similarly, as there was a good correlation between inhibition of IC and detection of 16S rRNA, we used the following equation to normalise our samples to the Cq of the IC:

Normalised 16S rRNA Cq = 16S rRNA Cq − [(IC Cq − 16.00) × 0.70979] (1)

0.70979 is the slope of the line, and it is a constant Figure 1A.

Next, we wanted to check the dynamic range of the MBL assay in tissues and establish a standard curve between the Cq of 16S rRNA determined via RT-qPCR and bacterial CFU/mL from agar. To do this, we spiked 10-fold serial dilutions of *M. tuberculosis* H37Rv (a range from 10^7^ to 10^0^ CFU/mL) into uninfected mouse tissues along with 50 ng of IC each time. RNA samples were extracted and quantified using RT-qPCR as before. Figure 1B demonstrates the standard curve of normalised Cq of 16S rRNA versus CFU/mL. Similarly, to the MBL assay from sputum samples, there is a good dynamic range of the assay on tissues between 10^8^ and 10^2^ CFU/mL (Figure 1B and Table 1). We used the following equation derived from the fitting of the standard curve to a continuous scale to determine the MBL values (CFU equivalents) of each sample:

MBL = (normalised 16S rRNA Cq − 34.04)/−3.6(2)

Having adapted the assay in tissues and validated its reproducibility and sensitivity, we then went on to trial the assay using infected mice treated with single drugs and drug combinations.

### 3.2. Detection of Variable Populations Using the MBL Assay

**Figure 2:**
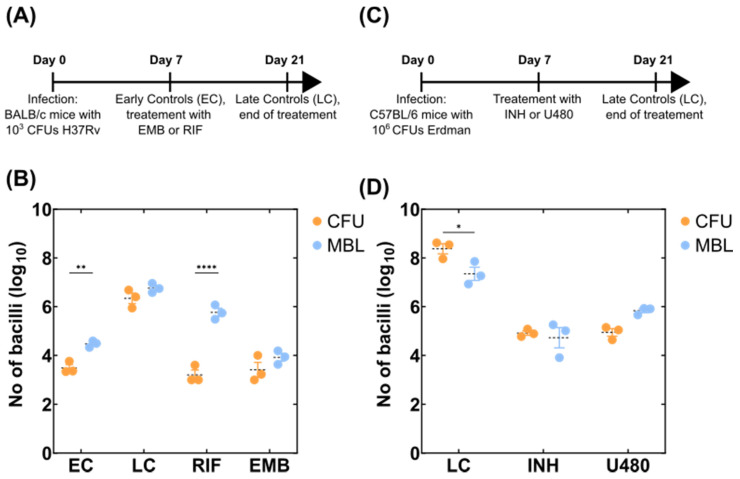
Comparison of MBL assay with CFUs from infected mouse tissues. (**A**) A timeline of the mouse experiment study 1 where the effect of RIF and EMB were tested. Each group contained three mice. (**B**) Bacterial load determined using CFUs on agar-based media versus MBL assay values determined using quantification of *M. tuberculosis* 16S rRNA using qPCR and normalised against the IC. A dotted black line indicates the mean of the group whereas the standard error is shown with the whiskers coloured according to each group. A two-way ANOVA test was used to determine statistical significance between different groups. *p* values are noted using asterisks (** *p* ≤ 0.01, **** *p* ≤ 0.0001). (**C**) A timeline of the mouse experiment study 2 where the effect of INH and U480 were tested. Each group contained three mice. (**D**) Bacterial load determined using colony forming units on agar-based media (CFUs) versus MBL assay values determined using quantification of *M. tuberculosis* 16S rRNA using qPCR and normalised against the IC. A dotted black line indicates the mean of the group whereas the standard error is shown with the whiskers coloured according to each group. A two-way ANOVA test was used to determine statistical significance between different groups. *p* values are noted using asterisks (* *p* ≤ 0.05).

In order to test the performance of the MBL assay to detect viable *M. tuberculosis* bacteria from animal experiments, we initially performed assays with single-drug treatments. Drugs were selected to represent different mechanisms of action to understand drug-specific variation on the MBL counts. Mice were infected with *M. tuberculosis* H37Rv (4.6 × 10^3^ CFU) and left untreated for 7 days (early controls: EC). Following the establishment of infection, drug treatment commenced for 14 days and then untreated controls (late controls: LC), as well as drug-treated mice (RIF, EMB), were sacrificed and their bacterial loads were enumerated via CFUs on agar-based media (right lung) and MBL assay via quantification of 16S rRNA (left lung) (Figure 2A). The bacterial load calculated for the untreated samples, both early and late controls, as well as on the mice treated with ethambutol were comparable when calculated by solid-agar CFU and the MBL assay (Figure 2B). Interestingly, there were different counts between CFUs and MBL assays in RIF treated animals, with MBL counting a higher bacterial load (median ~2.8 log_10_ higher).

In a separate experiment, we sought to investigate the ability of the MBL assay to determine the bacterial response in mice treated with either INH or sutezolid (PNU-100480 [U480]), an oxazolidinone that exhibits superior bactericidal activity against *M. tuberculosis* [23]. C57BL/6 mice were infected with 3.5 × 10^6^ CFU of *M. tuberculosis* Erdman. Mice were treated one week post-infection with 14 days of INH or sutezolid (Figure 2C). Untreated controls (LC, Figure 2D) from this experiment had a high bacterial load and a 1.6 log_10_ difference between CFUs and MBL counts. This could be due to the bacterial load being on the higher scale of the confidence intervals of the MBL assay and higher error based on the CFU counts at these dilutions. However, like the EMB treatment in the previous experiment, the INH group had also matched CFU and MBL counts. There was a small increase of around 0.5 log_10_ on the MBL compared to CFU for those treated with sutezolid (Figure 2D). Overall, in our hands, all treatment groups provided comparable results except for the RIF treatment group.

**Figure 3:**
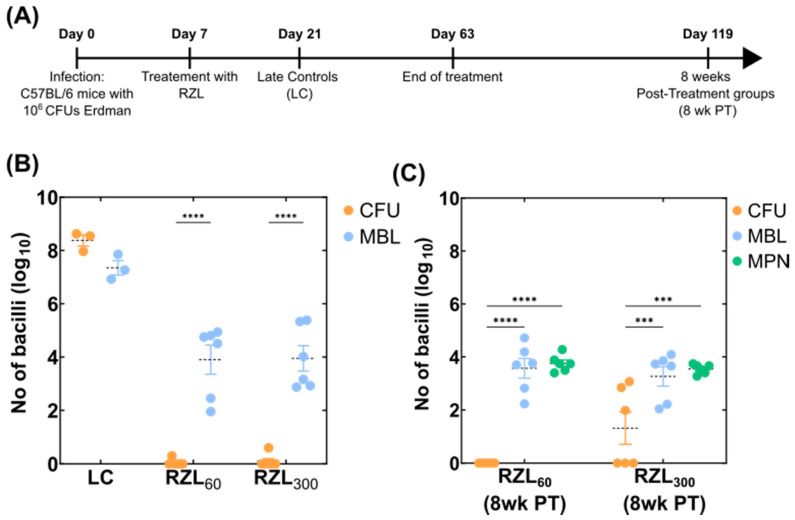
Application of the MBL assay on relapse models of infection. (**A**) A timeline of the mouse experiment study 2 where the effect of two RZL treatments were tested. Each group contained six mice whereas the LC group contained three mice. (**B**) Bacterial load determined using CFU on agar-based media versus MBL assay values determined using quantification of *M. tuberculosis* 16S rRNA using qPCR and normalised against the IC. A dotted black line indicates the mean of the group whereas the standard error is shown with the whiskers coloured according to each group. A two-way ANOVA test was used to determine statistical significance between different groups. *p* values are noted using asterisks (**** *p* ≤ 0.0001). (**C**) Bacterial load determined using CFU on agar-based media versus MBL assay values determined using quantification of *M. tuberculosis* 16S rRNA using qPCR and normalised against the IC versus MPN counts where the bacterial load was determined using serial dilutions in liquid-based media. A dotted black line indicates the mean of the group whereas the standard error is shown with the whiskers coloured according to each group. A two-way ANOVA test was used to determine statistical significance between different groups. *p* values are noted using asterisks (*** *p* ≤ 0.001, **** *p* ≤ 0.0001).

We then tested the efficacy of an experimental drug, rifalazil (RZL), a benzoxazinerifamycin that has a 64-fold lower MIC than RIF against many *M. tuberculosis* isolates and a half-life greater than 100 h [24]. Rifalazil achieves high intracellular concentrations and has no cytochrome P450 interactions, making it an attractive RIF replacement [25]. We treated C57BL/6 mice infected with 3.5 × 10^6^ CFU of *M. tuberculosis* Erdman with two different concentrations of RZL (either 60 or 300 mg/kg) for eight weeks (Figure 3A). Following treatment, bacterial load was quantified using CFUs and MBL assay as described. Both concentrations of RZL reduced the bacterial load over time with CFU counts showing complete sterilisation whereas the MBL assay showed only a 4-log_10_ reduction compared to the untreated control group (LC) (Figure 3B). Similarly, to RIF, RZL also produced a subpopulation of bacterial that were differentially culturable on agar media.

In this experiment, parallel groups of mice treated similarly with RZL were left untreated for eight weeks to investigate the sterilisation ability of this agent as well as forecast possible relapse episodes (Figure 3A). Interestingly, all CFU measurements showed complete clearance post eight weeks for the group that received 60 mg/kg of RZL whereas for the group that received 300 mg/kg of RZL, half of the samples were negative, and the other half measured bacterial loads ranging from 100 to 1000 CFUs. On the other hand, the MBL assay consistently measured an average mean of 10^4^ bacteria both for RZL60 and RZL300, indicating the possibility of persistent or differentially culturable organisms (Figure 3C). We further tested this hypothesis by calculating bacterial counts using the MPN assay where bacteria were grown in liquid media; the bacterial loads measured matched the ones of the MBL further suggesting that agar-based CFU counts could underrepresent the actual bacterial load present in tissues (Figure 3B).

**Figure 4:**
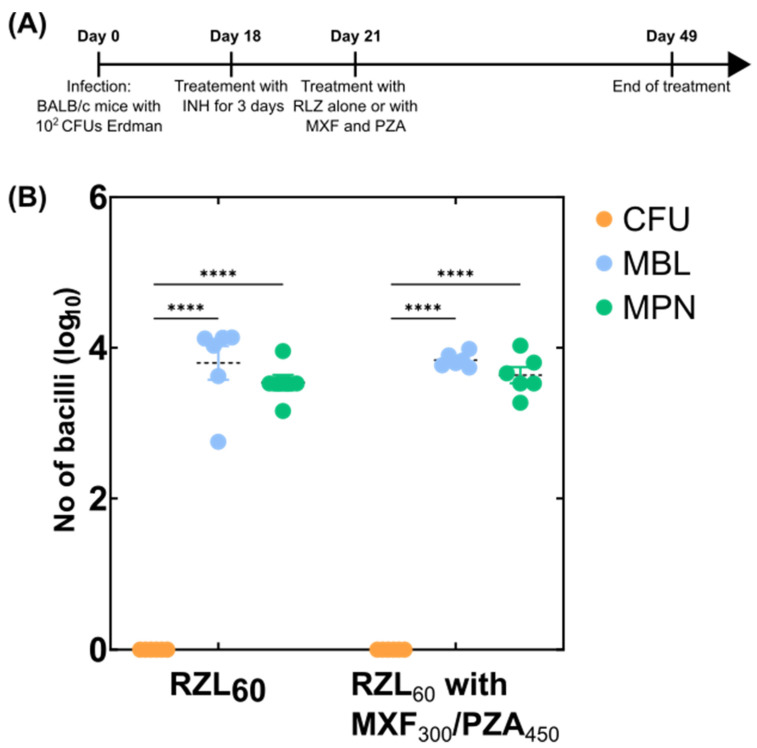
Sensitivity of MBL and MPN assays compared to CFUs on long drug treatment regimens. (**A**) A timeline of the mouse experiment study 3 where the effect of two RZL alone and in combination with MXF and PZA were tested. Each group contained six mice. (**B**) Bacterial load determined using CFU on agar-based media versus MBL assay values determined using quantification of *M. tuberculosis* 16S rRNA using qPCR and normalised against the IC versus MPN counts where the bacterial load was determined using serial dilutions in liquid media. A dotted black line indicates the mean of the group whereas the standard error is shown with the whiskers coloured according to each group. A two-way ANOVA test was used to determine statistical significance between different groups. *p* values are noted using asterisks (**** *p* ≤ 0.0001).

Lastly, we examined our MBL methodology on lung tissue from BALB/c mice infected with 2.6 × 10^2^ CFU of *M. tuberculosis* Erdman and treated with RZL (60 mg/kg) alone or in combination with moxifloxacin (MXF) at 300 mg/kg and pyrazinamide (PZA) at 450 mg/kg for four weeks (Figure 4A). Following treatment, we measured CFUs on agar, MBL count using RT-qPCR and MPN equivalents from liquid-based culture. Between the two treatments, we could not revive any viable bacteria using CFU on agar plates indicating a possible sterilising effect of both treatments and a good efficacy for RZL (Figure 4B). However, both the MBL and MPN assays showed a bacterial load with a median of about 10^4^ CFU equivalent present in all subjects indicating again the regimen had not cleared the bacteria from the mice completely as shown via CFU counts (Figure 4B).

## 4. Discussion

The purpose of this work is to ascertain whether the MBL assay, previously developed for enumeration of *M. tuberculosis* in sputum during treatment [18,19] was transferable to detection of *M. tuberculosis* from tissues during in vivo experiments. The MBL assay would then be able to provide a substitute for culture-based methods giving a rapid real-time readout without the need for culture. We validated the assay for mouse lung and spleen using spikes containing known numbers of *M. tuberculosis*. We then compared the MBL assay to both liquid and solid agar-based read-outs using lungs from animals challenged with *M. tuberculosis* and subsequently treated with different drugs.

Experiments testing mouse lung spiked with *M. tuberculosis* were used to validate the MBL assay for tissues and supported a robust readout similar to that found for sputum with a wide range of detection for bacterial loads [18].

Treatment for tuberculosis is protracted but shortening treatment with the current regimens for drug-sensitive TB leads to an unacceptably high relapse rate [26,27,28] suggesting that there are populations of bacteria that are difficult to kill [29,30]. Our previous study compared culture-based methods and the MBL assay using different drug combinations and ascertained that the MBL assay was able to detect a subpopulation of bacteria not detected by culture [8]. This difference in sub-populations was also confirmed here in the RZL treatment group and at subsequent follow-up (Figure 3) where the bacteria load remained similar when counted using MBL or MPN assays but were at the limit of detection using agar-based CFU counts. This outcome further supports the hypothesis that the MBL assay indeed captures a differentially culturable population that is perhaps under stress, dormant and it is not able to grow on solid media. Moreover, both MBL and MPN assays captured bacterial loads that were stable over the 8-week post-treatment period indicating the adaptive immunity of these mice being able to suppress the infection further. In addition, we showed here the applications that the MBL assay could have in developing and ranking novel drug regimens for prioritisation towards further clinical trials. The MBL assay can, in real time, provide quantification of bacilli present following therapy as an indication of sterilisation activity of the various treatments under investigation, allowing for quick adaptations of animals and treatment options. This could further accelerate the preclinical stages of drug development and the selection of potent regiments for downstream investigation. A similar method where the ratio of the rRNA synthesis (RS ratio is the ratio of external transcribed spacer 1 (ETS1) to 23S rRNA) has been described in *M. tuberculosis* to be able to predict regimens with better sterilising activity indicating the relevance that the ribosomal RNA can have as a biomarker for animal models of TB in the place of CFUs [31]. In the same study, the activity of rifampicin reduced the RS ratio indicating strong sterilisation that also matched the decline of CFU counts [31]. In our case, the MBL assay detected a higher number of bacterial load present highlighting the fact that even though there is killing associated with RIF treatment, substantial numbers of bacteria in these samples were differentially culturable bacteria in accordance with previously published results that could have an important role for future treatment success [29,32,33].

The study presented here examined the contribution of single-drug treatments and combination regimens to inform which drugs are responsible for the discrepancies between culture-based measurements and the MBL assay. Our data suggested that comparable readouts are obtained for single drug treatments with isoniazid (INH) ethambutol (EMB) and sutezolid comparing our newly validated MBL assay for tissues with CFU on solid agar. In contrast, when rifampicin (RIF) and rifalazil (RZL) were administered to mice, the MBL assay always detected significantly higher readouts in comparison to bacteria detected using solid culture. This data supports our previous finding for RIF-containing regimens [8] and suggests it is not attributable to all the drugs in the regimen but to ones targeting RNA transcription. As rifampicin targets RNA replication, we thought that the MBL signal (i.e., 16S rRNA) might be lower and thus provide an underestimation of the actual bacterial load; however, we observed the contrary in our study. The effect of rifampicin on the ribosome may prevent the growth of a single bacteria into a visible colony on an agar plate due to slowed translation but these bacteria are still viable.

When the MPN method was included in the analysis, it gave further support to the MBL assay detecting a population of bacteria that were still viable rather than residual rRNA from non-viable organisms. In vitro studies have begun to characterise these populations [34,35,36]. Our data further support the existence of these populations in mouse lungs. MBL and MPN data correlate well supporting the utility of the MPN assay towards bacterial enumeration from tissues and the advantage of liquid-based assays.

In summary, we have shown that using the MBL assay we were able to measure populations of bacteria that are not culturable on solid agar and validated that these were indeed viable cells using liquid-based media. In addition, our data highlight the potential use of the MBL assay to predict relapses from animal experiments in real time. Since the MBL assay uses molecular methods, results can be obtained within 24 h post-mortem regardless of the starting bacterial burden. The MBL assay has the potential to decrease the readout time of data from animal experiments and represents a valuable tool for tuberculosis drug discovery and preclinical drug development.

## Figures and Tables

**Table 1:** Standard curve range of Cq values specified for each concentration of H37Rv. Values are taken from the data shown in Figure 1B.

	Bacilli mL^−1^ Tissue							
	10^8^	10^7^	10^6^	10^5^	10^4^	10^3^	10^2^	<10^2^
Mean (Cq)	6.0	8.0	14.6	17.3	22.3	24.9	27.6	28.7
± SD	0.9	0.6	1.0	0.9	1.2	0.9	1.1	-

## Data Availability

All data associated with this study are openly available at https://doi.org/10.5522/04/19175153.v2 (accessed on 29 December 2021).
